# Morbidity and Risk of Subsequent Diagnosis of HIV: A Population Based Case Control Study Identifying Indicator Diseases for HIV Infection

**DOI:** 10.1371/journal.pone.0032538

**Published:** 2012-03-05

**Authors:** Ole S. Søgaard, Nicolai Lohse, Lars Østergaard, Gitte Kronborg, Birgit Røge, Jan Gerstoft, Henrik T. Sørensen, Niels Obel

**Affiliations:** 1 Department of Infectious Diseases, Aarhus University Hospital, Skejby, Denmark; 2 Department of Clinical Epidemiology, Aarhus University Hospital, Aarhus, Denmark; 3 Department of Infectious Diseases, Copenhagen University Hospital, Hvidovre, Denmark; 4 Department of Infectious Diseases, Kolding Hospital, Kolding, Denmark; 5 Department of Infectious Diseases, Rigshospitalet, Copenhagen, Denmark; 6 Department of Epidemiology, Boston University School of Public Health, Boston, Massachusetts, United States of America; University of Nebraska Medical Center, United States of America

## Abstract

**Background:**

Early identification of persons with undiagnosed HIV infection is an important health care issue. We examined associations between diseases diagnosed in hospitals and risk of subsequent HIV diagnosis.

**Methods:**

In this population-based case control study, cases were persons with incident HIV infection diagnosed in Denmark between 1 January 1995 and 1 June 2008. Risk-set sampling was used to identify 19 age- and gender-matched population controls for each HIV case, using the HIV diagnosis date as the index date for both cases and controls. Prior hospital diagnoses obtained from Danish medical databases were first categorized into 22 major disease categories (excluding AIDS-defining diseases except tuberculosis) and then subdivided into 161 subcategories, allowing us to examine specific diseases as potential HIV indicators by conditional logistic regression.

**Results:**

The study included 2,036 HIV cases and 35,718 controls. Persons with the following disease categories had a high risk of HIV diagnosis during the subsequent 5-year period: sexually transmitted infections and viral hepatitis (adjusted odds ratio [aOR] = 12.3, 95% CI: 9.60–15.7), hematological diseases (aOR = 4.28, 3.13–5.85), lower respiratory tract infections (aOR = 3.98, 3.14–5.04)), CNS infections (aOR = 3.44, 1.74–6.80), skin infections (aOR = 3.05, 2.47–3.75), other infections (aOR = 4.64, 3.89–5.54), and substance abuse (aOR = 2.60, 2.06–3.29). Several specific diseases were associated with aORs >20 including syphilis, hepatitis A, non “A” viral hepatitis, herpes zoster, candida infection, endocarditis, thrombocytopenia, and opioid abuse.

**Conclusions:**

Targeted testing for HIV in patients diagnosed with diseases associated with HIV may lead to earlier treatment and thereby reduced morbidity, mortality and HIV transmission.

## Introduction

Despite three decades of concerted effort, the HIV epidemic remains a tremendous public health challenge in both low- and high-income countries [1], [2]. Many individuals newly diagnosed with HIV present at a late stage of the disease with severe immune depletion, resulting in delayed initiation of antiretroviral therapy (ART) which worsens their prognosis [3] and increases further transmission of HIV [4], [5], [6]. Recently, the European Center for Disease Control and Prevention's Dublin Declaration Progress Report 2010 concluded that “the rates of late diagnosis remain unacceptably high” in Europe and Central Asia [7]. Thus, intensified HIV testing and treatment has been advocated to lower the prevalence of undiagnosed HIV infection and to control the HIV epidemic [8], [9].

The U.S. Centers for Disease Control and Prevention (CDC) recommend routine HIV testing for all persons under age 65 who come into contact with the health care system [10]. While this approach may be cost-effective in areas with high HIV prevalence [11] and does prolong life expectancy [3], alternative approaches such as targeted HIV testing may be more appropriate in other settings with lower HIV prevalence or different health care systems [2].

Targeted HIV testing based on risk groups (e.g. injecting drug users [IDU] [12]), presence of AIDS-defining illnesses, or coming from a high-HIV prevalence country is practised in most European countries [13]. However, many opportunities for HIV testing are missed by the health care system prior to HIV diagnosis [14]. In 2007, the pan-European initiative “HIV in Europe” recommended that targeted testing based on the presence of diseases associated with HIV, so-called indicator diseases [15], should be developed as an additional tool to guide targeted HIV testing. HIV indicator diseases may be a result of individual risk behavior or coexisting HIV infection. Several studies have identified indicator diseases within a narrow spectrum of conditions [16], [17], [18], [19], but to date there has been no comprehensive study of HIV indicator diseases. In the absence of adequate data, guidance is based mainly on expert opinion [15]. There is substantial information regarding the prevalence of indicator diseases in the HIV-infected population. In contrast, the relative risk of HIV among patients with indicator diseases remains poorly described. The goal of this study was to delineate medical conditions that identify individuals at increased risk of subsequent HIV diagnosis.

## Methods

### Ethics statement

The Danish Data Protection Agency approved the establishment of the cohort and the linkage between the four registries in this study (J.-no. 2008-41-1781). The study was not subject to approval by the ethics committee because data collection did not involve direct patient contact and all data was fully de-identified.

We conducted a population-based nested case control study among persons with and without an incident HIV diagnosis in Denmark. The adult population of Denmark is 4.3 million with an estimated HIV infection prevalence of 0.09% [20]. The Danish healthcare system provides free, tax-supported medical care for all residents, including antiretroviral treatment of HIV.

### Data sources

The civil registration system (CRS) number, assigned at birth, uniquely identifies each person living in Denmark since 1968 and is used for personal identification in all Danish administrative and medical databases. We used this unique 10-digit CRS-number to link data among the following registries: *The Danish HIV Cohort Study* (DHCS) is a prospective, open, nationwide, population-based cohort of all HIV-infected individuals receiving care in Danish HIV clinics since 1 January 1995 [20]. The study is ongoing, with continuous enrolment of newly diagnosed patients. *The Danish Civil Registration System* records demographic information, vital status, and immigration and emigration dates for all Danish citizens beginning in 1967 [21]. *The Danish National Registry of Patients* (DNRP) contains information on all patients discharged from Danish hospitals since 1977 [22]. It includes diagnoses coded by the treating physician according to the *International Classification of Diseases*, 8th revision (ICD-8) up to the end of 1993 and according to the 10th revision (ICD-10) thereafter. The registry covers public as well as private hospitals and so constitutes a virtually complete population-based database. *The Danish Cancer Registry* (DCR) has recorded all incident cancers in Denmark since 1943, classifying cancers registered after 1977 according to ICD-10 [23].

### Study population

#### Cases

Cases were identified from DHCS and included all individuals who (I) were diagnosed with HIV between 1 January 1995 and 1 June 2008; (II) were at least 16 years of age on the date of HIV diagnosis; and (III) were living in Denmark for at least 5 years prior to HIV diagnosis. The index date for HIV cases was defined as the date of first positive HIV test as registered in the DHCS.

#### Population controls

Controls not diagnosed with HIV were identified from the CRS using incidence density sampling, which involves matching each case to a sample of those who are at risk at the time of case occurrence [24]. To ensure sufficient statistical power to detect differences in the occurrence of rare events, we sampled for each case 19 random age- and gender-matched population controls that were alive on the HIV diagnosis date of their respective case. The date of HIV diagnosis/sampling constituted the *index date* for both cases and controls. Controls who, according to the CRS, had not been living in Denmark for at least 5 years prior to the index date were excluded. Hence, all study subjects were living in Denmark during the 5-year period prior to their index date and therefore at risk of both exposure and outcome.

### Identification of hospital diagnoses and grouping of disease categories

For all study subjects we extracted hospital diagnoses from outpatient contacts and hospital stays from the DNRP and DCR, up to the day prior to the index date. ICD-10 codes were the primary source for grouping diseases. We defined 22 disease categories of interest according to the type and anatomical location of the disease (Table S1). ICD-8 codes were translated to the corresponding ICD-10 disease categories and first-time diagnoses were assigned to the appropriate category. In addition, a total of 161 subcategories were created for the 22 disease categories, allowing us to examine specific diseases as potential HIV indicators (Table S1). Except for tuberculosis, we excluded AIDS-defining diseases, because their association with HIV infection is well established [25].

### Statistical analyses

For cases and controls, we tabulated gender, age (16–39 years, 40–49 years, 50–59 years, and 60+ years), and hospital contact(s) in the 5-year period prior to the index date for each of the 22 disease categories (yes/no). For cases, the following variables were also included: race, most likely mode of HIV acquisition, presence of AIDS [25] at diagnosis, first CD4^+^ cell count, and HIV RNA measurement (within 180 days of HIV diagnosis). Frequencies and percentages were computed for all variables.

### Conditional logistic regression analyses

For each of the 22 disease categories, conditional logistic regression analysis was used to estimate odds ratios (OR), which is an unbiased estimate of the incidence rate ratio (IRR) for subsequent HIV diagnosis [24]; unadjusted ORs as well as ORs adjusted for the remaining 21 disease categories were estimated. Observation data were subsequently stratified into three time periods prior to the index date (less than 1 year, 1–2 years, and 3–5 years,) to explore changes in ORs in the years following a given disease or disease category. We used first-time diagnoses registered within each time stratum for a given disease/disease category. Adjusted ORs were calculated for each disease category and time period (adjusted for the remaining 21 disease categories). To identify specific HIV-indicator diseases, we explored risk estimates for the 161 specific subcategories within each of the 22 disease categories. We computed both unadjusted odds ratios for each subcategory and odds ratios adjusted for other diseases within the same disease category (Table S1). In all analyses, only first-time diagnoses for a given disease/disease category were utilized.

## Results

We identified 2,363 individuals diagnosed with HIV between 1 January 1995 and 1 June 2008 and 38,684 controls. Of these, 327 cases and 2,966 controls were excluded because they immigrated to Denmark less than 5 years prior to their index date. Thus, a total of 2,036 cases and 35,718 controls were included resulting in an average of 17.5 controls per case.

In the 5 years prior to their index date, there were a total of 138,416 hospital contacts for both cases and controls, of which 34,520 represented a first-time diagnosis for one of the 22 disease categories. Table 1 shows characteristics of cases and controls on their index date. In the 5 years preceding the index date, 69.8% of cases (1,421 of 2,036 cases) and 53.6% of controls (19,148 of 35,718 controls) had at least one hospital contact for one of the 22 disease categories delineated below.

**Table 1:** Baseline characteristics^a^ of HIV cases and HIV-uninfected controls.

		Cases	Controls
		(n = 2,036)	(n = 35,718)
Sex, n (%)			
	Female	380 (18.7)	6,555 (18.4)
	Male	1,656 (81.3)	30,819 (81.6)
Age at diagnosis, n (%)			
	16–39 years	1,122 (55.1)	18,899 (52.91)
	40–49 years	510 (25.1)	9,266 (25.9)
	50–59 years	290 (14.2)	5,410 (15.2)
	60+ years	114 (5.6)	2,143 (6.0)
Hospital contact, n (%)^b^			
	Yes	1,421(69.8)	19,148 (53.6)
	No	615 (30.2)	16,570 (46.4)
Race, n (%)			
	Caucasian	1,798 (88.7)	–
	Black	119 (5.9)	–
	Asian	55 (2.7)	–
	Inuit	19 (0.9)	–
	Other	36 (1.8)	–
Mode of HIV exposure, n (%)			
	MSM	975 (47.9)	–
	Heterosexual	722 (35.5)	–
	IDU	210 (10.3)	–
	Other	49 (2.4)	–
	Unknown	80 (3.9)	–
AIDS at diagnosis, n (%)			
	No	1,847 (90.7)	–
	Yes	189 (9.3)	–
First CD4^+^ count (cells/µL)^c^			
	<350	999 (49.1)	–
	≥350	829 (40.7)	–
	*missing*	208 (10.2)	
First HIV RNA measurement (copies/mL)^c^			
	≤10^4^	343 (16.9)	–
	>10^4^ & ≤10^5^	622 (30.6)	–
	>10^5^	660 (32.4)	–
	*missing*	411 (20.2)	

a Baseline was defined as the index date which was the date of HIV diagnosis for cases and their corresponding controls (matched on the day the case was diagnosed with HIV).

b In the 5 years prior to the index date for at least one of the 22 disease categories in Table S1.

c Within 180 days of HIV diagnosis. The table shows data on HIV cases and HIV-uninfected controls with at least 5 years of continuous observation prior to the index date. MSM, men who have sex with men; IDU, injection drug use.

### Disease category and risk of subsequent HIV diagnosis

**Figure 1:**
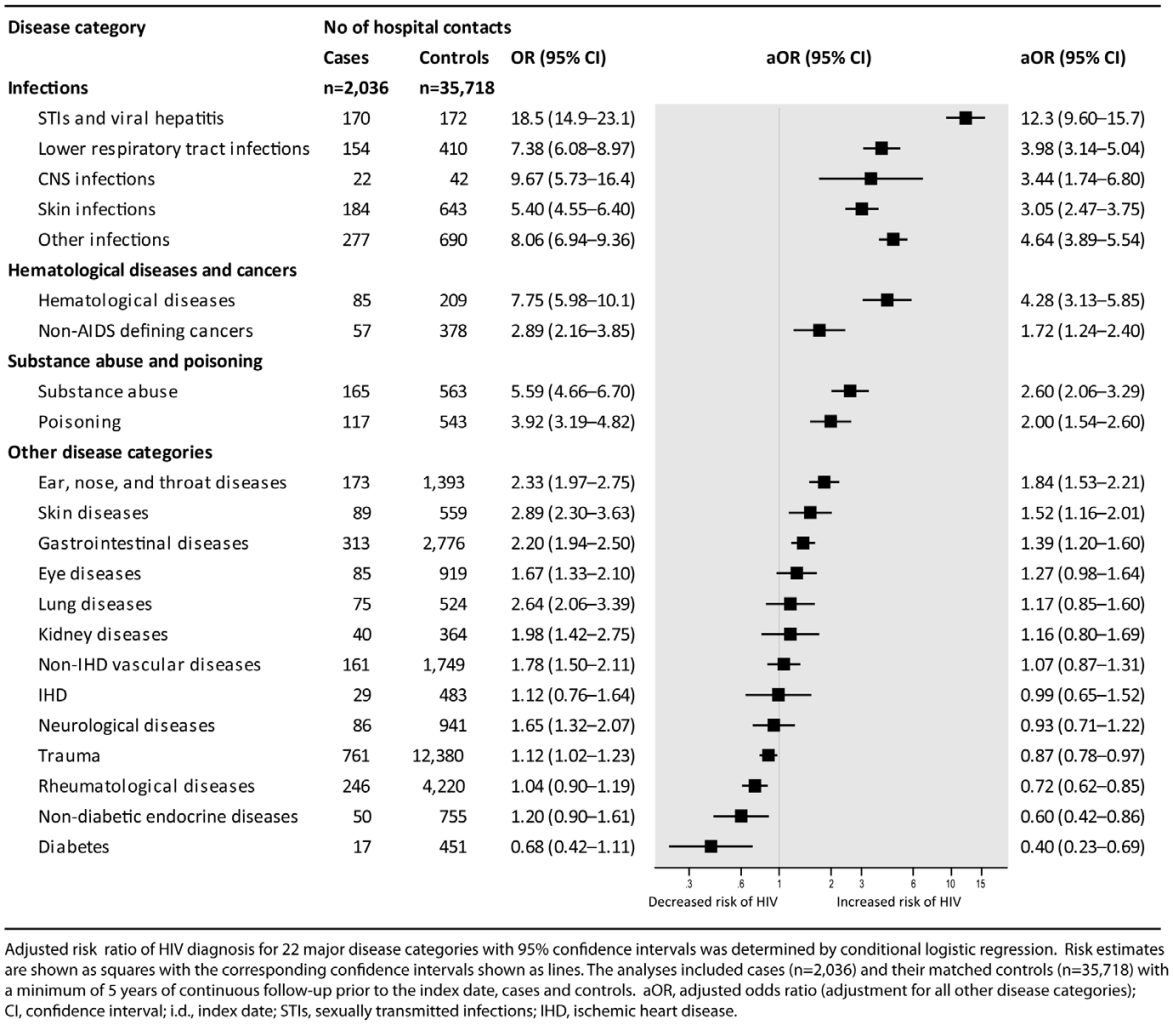
Association between subsequent risk of HIV diagnosis and hospital contact for 22 major disease categories.

Several disease categories were associated with an increased risk of HIV diagnosis during the following 5-year period (Figure 1). Persons diagnosed a disease in the category “sexually transmitted infections (STIs) and viral hepatitis” had the highest risk of subsequent HIV diagnosis (adjusted OR [aOR] = 12.3, 95% confidence interval [CI], 9.60–15.7). Also persons with infections (lower respiratory tract infections, CNS infections, skin infections, and other infections), hematological diseases, non-AIDS defining cancers, substance abuse, poisoning, ear, nose, and throat diseases, skin diseases, and gastrointestinal diseases had higher risk of HIV diagnosis during the following 5 years. Seven disease categories were not associated with subsequent HIV diagnosis: eye diseases, kidney diseases, lung diseases, ischemic heart disease (IHD), non-IHD vascular diseases, neurological diseases, and trauma. A decreased risk of subsequent HIV diagnosis was found for persons with rheumatological diseases (aOR = 0.72, 95% CI:0.62–0.85), non-diabetes endocrine diseases (aOR = 0.60, 95% CI:0.42–0.86), and diabetes (aOR = 0.40, 95% CI:0.23–0.69).

### Time trends in risk of HIV diagnosis following hospital contact

**Figure 2:**
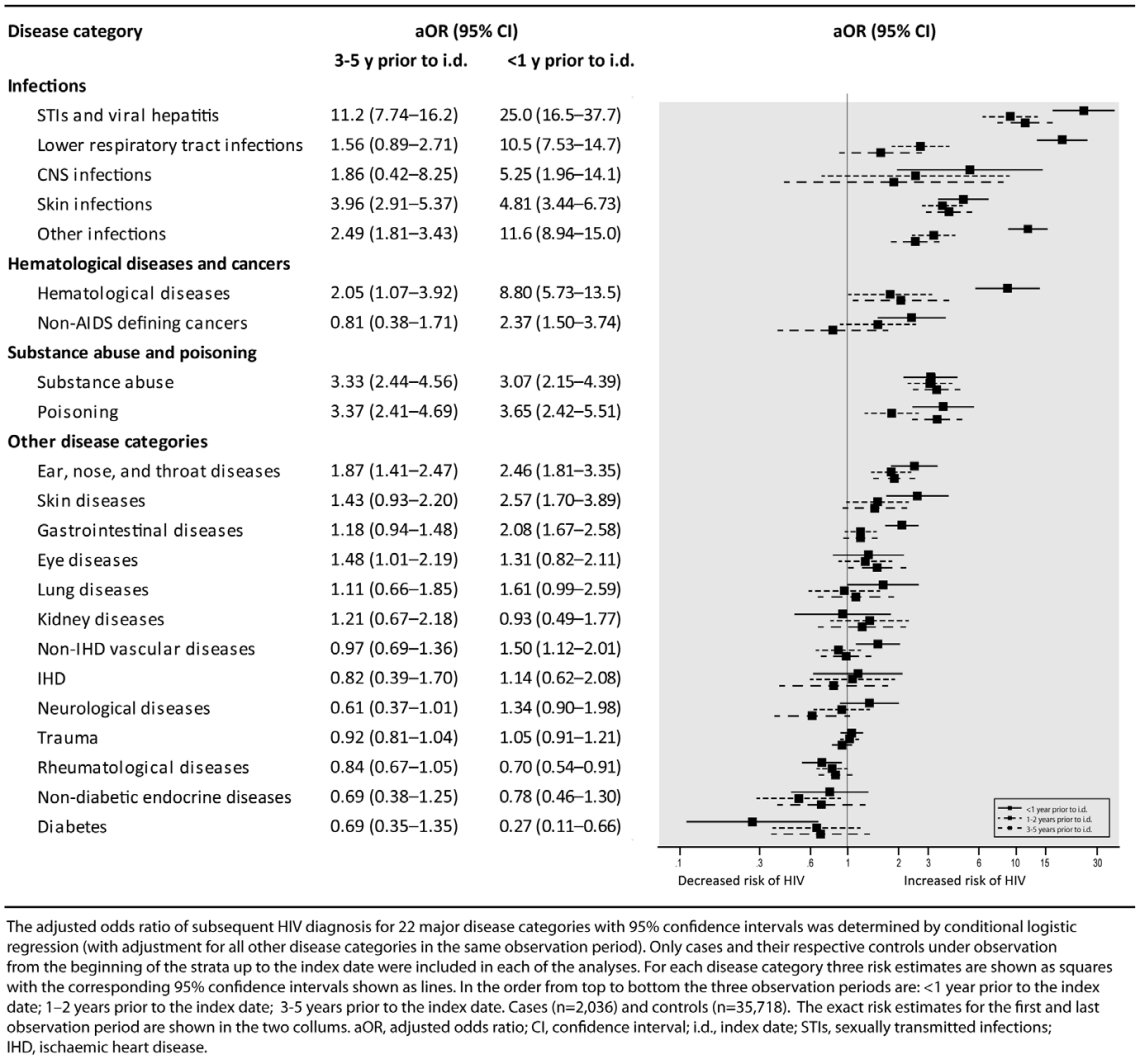
Association between risk of subsequent HIV diagnosis and time of hospital contact.

Figure 2 shows the relative risk of subsequent HIV in 3 time periods: <1 year, 2–3 years, and 3–5 years after each of the 22 disease categories. The time trends can be categorized in four main groups: 1) no time trend (eye diseases, lung diseases, kidney diseases, IHD, neurological diseases, trauma, rheumatological diseases, non-diabetic endocrine diseases, and diabetes); 2) A highly increased risk of HIV in the first year after diagnosis and no or only a slightly increased risk thereafter (hematological diseases, non-AIDS malignancy, skin diseases, gastrointestinal diseases, and non-IHD vascular diseases); 3) gradually decreased risk of HIV over time (lower respiratory tract infections, CNS infections, skin infections, other infections, and ear, nose, and throat diseases); 4) persistently increased risk both in the short and the long term (STIs and viral hepatitis, substance abuse, and poisoning).

### Specific HIV-indicator diseases

To determine whether any specific diseases were strong indicators of subsequent HIV diagnosis, we further divided the 22 disease categories into 161 subcategories. Table 2 reports risk estimates for subcategories associated with at least a 3-fold elevated risk of subsequent HIV diagnosis (all 161 subcategories shown in Table S2). We found strong associations between all groups of STIs and viral hepatitis and subsequent HIV diagnosis. Several other specific groups of infectious diseases, including meningitis, herpes zoster, endocarditis, and malaria, were closely associated with later HIV diagnosis. Among hematological diseases and cancers: thrombocytopenia, anemia, lymphadenitis, non-AIDS defining lymphomas, and secondary and unspecified malignant neoplasm of lymph nodes were associated with later HIV diagnosis. Genital cancers were not. In the group of gastrointestinal diseases, diseases of the oral cavity, liver diseases, and fissures/abscesses of the anal cavity were strongly associated with later HIV diagnosis. Among skin diseases, seborrhoeic dermatitis was associated with increased risk of HIV diagnosis. Ischemic heart disease, including myocardial infarction (aOR = 0.81, 95%:0.39–1.69) and other cardiovascular diseases, were not associated with later HIV diagnosis, with the notable exception of thrombophlebitis (aOR = 5.29, 95% CI:3.51–7.96).

**Table 2:** Specific diseases (diagnosed in the 5 years before the index date) associated with at least 3-fold elevated risk of subsequent HIV diagnosis.

Disease category	Specific disease	Cases n = 2,036	Controls n = 35,718	aOR (95% CI)^a^
**Infections**				
STIs and viral hepatitis	Syphilis	15	2	94.7 (20.9–429)
STIs and viral hepatitis	Hepatitis A	14	4	41.6 (11.7–148)
STIs and viral hepatitis	Non “A” viral hepatitis^b^	77	55	23.6 (16.5–33.7)
STIs and viral hepatitis	Anogenital herpes simplex	7	10	12.7 (4.65–34.8)
STIs and viral hepatitis	Condyloma	55	95	8.99 (6.32–12.8)
STIs and viral hepatitis	Other STIs	18	12	14.8 (6.35–34.6)
Lower respiratory tract infections	Unspecified pneumonia	126	290	7.56 (6.03–9.48)
Lower respiratory tract infections	Pneumococcal pneumonia	9	14	4.33 (1.63–11.5)
Lower respiratory tract infections	Influenza and viral pneumonia	9	43	3.21 (1.51–6.81)
CNS infections	Bacterial meningitis	5	9	14.7 (5.63–38.1)
CNS infections	Viral meningitis or encephalitis	9	25	6.33 (2.90–13.8)
CNS infections	Other CNS infections	9	10	5.51 (1.60–19.0)
Skin infections	Abscess, furuncle, carbuncle	137	409	5.15 (4.17–6.35)
Skin infections	Fungal skin infections	3	11	4.41 (1.18–16.5)
Skin infections	Erysipelas	25	76	3.92 (2.39–6.45)
Skin infections	Other skin infections	45	95	5.29 (3.56–7.86)
Other infections	Herpes zoster	22	8	33.7 (14.3–79.6)
Other infections	Candida infection^c^	40	22	25.5 (14.6–44.6)
Other infections	Endocarditis	11	7	23.2 (8.71–61.9)
Other infections	Tuberculosis and other mycobacterial infections	24	21	15.2 (7.99–29.1)
Other infections	Malaria	10	12	9.53 (3.86–23.5)
Other infections	Mononucleosis	11	19	8.64 (4.04–18.5)
Other infections	Lymphangitis	5	8	7.88 (2.40–25.9)
Other infections	Unspecified viral illness	23	44	7.87 (4.56–13.6)
Other infections	Sepsis	23	34	4.90 (2.52–9.52)
Other infections	Infectious gastroenteritis	50	216	3.48 (2.49–4.87)
Other infections	Other types of infection	67	182	4.77 (3.46–6.56)
**Hematological diseases and cancers**				
Hematological diseases	Thrombocytopenia	15	10	24.0 (10.5–54.7)
Hematological diseases	Unspecified anemia	24	44	7.26 (4.19–12.6)
Hematological diseases	Lymphoma	18	43	5.83 (3.22–10.5)
Hematological diseases	Aplastic and other specified anemias	7	17	4.58 (2.38–8.79)
Hematological diseases	Lymphadenitis	13	42	3.44 (1.42–8.30)
Hematological diseases	Nutrition deficiency anemia	7	27	3.11 (1.11–8.70)
Hematological diseases	Other hematological diseases	5	18	4.30 (1.54–12.0)
Non-AIDS defining cancers	Secondary and unspec. malignant neoplasm of lymph nodes	11	22	6.74 (3.14–14.5)
**Substance abuse and poisoning**				
Substance abuse	Substance abuse opioids	69	17	43.5 (24.6–76.8)
Substance abuse	Substance abuse other	51	55	6.54 (4.07–10.5)
Drug poisoning	Narcotics and hallucinogens	47	43	11.2 (7.08–17.8)
**Other disease categories**				
Ear, nose, and throat diseases	Other acute upper respiratory tract infection	10	30	5.02 (2.38–10.6)
Ear, nose, and throat diseases	Chronic disease of tonsils and adenoids	43	129	4.95 (3.45–7.09)
Skin diseases	Seborrhoeic dermatitis	9	8	11.8 (4.30–32.6)
Gastrointestinal diseases	Fissure/abscess of anal and rectal regions	50	202	4.35 (2.87–6.61)
Gastrointestinal diseases	Liver diseases	31	103	4.06 (2.27–7.25)
Gastrointestinal diseases	Disease of salivary glands, oral mucosa, tongue and lips	15	58	3.97 (2.88–5.48)
Lung diseases	Respiratory disease principally affecting the interstitium	7	13	9.22 (3.63–23.4)
Lung diseases	Lung abscess/empyema without pneumonia	5	11	6.42 (2.16–19.1)
Lung diseases	Pneumothorax	8	40	3.25 (1.98–5.35)
Lung diseases	Other lung diseases	21	95	3.00 (1.37–6.55)
Non-IHD vascular diseases	Thrombophlebitis	34	106	5.29 (3.51–7.96)
Neurological diseases	Facial nerve disorder	8	31	3.04 (1.46–6.35)
Neurological diseases	Polyneuropathy	9	47	4.52 (2.07–9.85)
Rheumatological diseases	Infectious arthropathy	14	75	3.18 (1.76–5.74)

a Adjusted for other diseases within the same disease category (Table S1).

b Diagnosis of non-A hepatitis coded by ICD-10 were evenly distributed between hepatitis B and C with no significant difference in their association to risk of subsequent HIV diagnosis.

c The subcategory *candida infection* primarily consisted of “oral candidiasis” but other types of candidiasis also occurred albeit at too low frequencies to be analyzed seperately. Risk estimates for all 161 subcategories are shown in Table S2. aOR, adjusted odds ratio; CI, confidence interval; STIs, sexually transmitted infections; IHD, ischemic heart disease.

Among fourteen individuals diagnosed with hepatitis A infection, 12 (85.7%) were men who had sex with men (MSM). Five (50%) of ten cases with malaria were non-Caucasian immigrants. In 62 of 68 cases (91.2%) with opioid abuse-related diagnoses, IDU was registered as the mode of HIV transmission.

First-time diagnoses for the 52 diseases in Table 2 represented 3,257 (1.9%) of all 138,416 hospital contacts registered in the 5 years prior to the index date. In the HIV cohort of 2,036 individuals, 782 (38.4%) had at least one hospital contact for an indicator disease, while this occurred for only 2,475 (6.9%) of 35,718 controls. For the 1,826 cases not reporting IDU as mode of HIV infection, 613 (34.1%) had a first-time hospital contact for diseases listed in Table 2.

## Discussion

We found that HIV indicator diseases identified over one-third of all individuals who would become diagnosed with HIV in the subsequent 5-year period. Recognition of these HIV indicator diseases could aid healthcare personnel in identifying individuals at increased risk of undiagnosed HIV. The time-dependent association between the date of diagnosis of some disease categories and HIV diagnosis suggests that for several categories repeated (e.g. yearly) HIV testing is advisable.

To our knowledge, this is the first population-based study conducted to identify HIV indicator diseases across all disease categories. While some indicator diseases we identified were previously recognized [15], [16], [17], [26], the size of our study population and completeness of hospitalization data allowed us to provide risk estimates with high statistical precision for the majority of disease groups. Our results thus can be used to guide strategies for targeted HIV testing in the hospital settings.

Our study also had some limitations. As in other observational HIV cohort studies, an unknown proportion of people with HIV may have died without being diagnosed with the condition. Thus, we may have underestimated the relative risk of subsequent HIV diagnosis after life-threatening conditions such as cancer [26]. Additionally, we had access only to hospital diagnoses, which may have caused us to underestimate the occurrence of diseases diagnosed outside the hospital system, such as syphilis, fungal skin infections, and herpes zoster. If patients at risk of HIV are more prone to be diagnosed in a hospital setting this will lead to a potential overestimation of the predictive value of some HIV predictor diseases if compared with those from a non-hospital setting. Another potential shortcoming is inaccuracies in diagnoses reported to national hospital databases. However, the positive predictive value of registry diagnoses (i.e. the proportion of subjects with a given registry diagnosis which is correct when compared to medical records) is generally high (70%–99%) [22], [27]. Furthermore, risk of subsequent HIV diagnosis may be elevated if a given disease diagnosis increases the likelihood of HIV testing, regardless of its actual association with HIV infection. This phenomenon would produce a close association between the timing of the disease and HIV diagnoses. Although persons with some disease categories had increased risk of HIV in the first year thereafter (Figure 2), the increased risk of subsequent HIV diagnosis was also observed more than 1 year after the disease. Hence, this phenomenon could only have a moderate effect on the 5-year estimates. Finally, it should be noted that due to our use of hospital diagnoses, we were unable to identify clinical features that may further enhance the predictive value of given indicator diseases (e.g. “florid or hard to treat” fungal skin infection).

HIV indicator diseases can be grouped into three major types. First, disease manifestations of acute HIV infection (e.g. acute viral illness or lymphadenitis); second, diseases associated with coexisting HIV infection (e.g. herpes zoster or thrombocytopenia); and third, diseases associated with behavior that increases the risk of acquiring HIV (e.g. hepatitis and opioid abuse). The latter group is highlighted in Figure 2, which shows that disease categories related to individual risk behaviors (e.g. STIs and viral hepatitis) are associated with an elevated risk that remained constant over time. Other disease categories which may be related to coexisting HIV, such as respiratory infections, hematological diseases, and non-AIDS-defining cancers, showed a highly increased risk of HIV in the first year after the diagnosis. The close connection between the date of hospital contact and date of HIV diagnosis suggests that the hospital contacts led to subsequent HIV testing as part of the recommended medical work-up (e.g. following lymphoma diagnosis) [15]. Among specific HIV indicator diseases, acute hepatitis A virus (HAV) infection was associated with high risk of subsequent HIV diagnosis. The strong association between acute HAV infection and MSM suggests that HAV infection is a proxy for high-risk sexual behavior in our study population [28]. Our data also indicate that endocrine and rheumatological diseases (except infectious arthropathy) are associated with decreased risk of HIV which may be related to low-risk sexual behavior among individuals with these chronic diseases [29]. Factors associated with a specific behaviour or transmission risk (such as MSM, IDU, and migration) may represent potential confounders. Information on these risk factors were not available for controls and thus, could not be adjusted for in the analyses. However, the purpose of the present study was to identify indicator diseases regardless of their causal or non-causal relationship to HIV infection. Therefore, our estimates represent the risk of a given disease compared to a representative sample of a background population of mixed race and sexual orientation. Our study only included first-time diagnosis and we did not assess the effect of repetitively diagnoses (e.g. pneumonia). Repetitive diagnoses of indicator diseases in an individual is most likely associated with even higher risk of undiagnosed HIV, and thus should prompt immediate HIV testing.

Targeted HIV testing is practiced in many countries [2], [13], [15] and may be more cost-effective than universal HIV testing in low HIV prevalence regions like Denmark [30], [31]. However, until now the lack of a thorough delineation of HIV indicator diseases has markedly reduced the efficacy of targeted HIV testing [2]. Another barrier to HIV screening is the acceptance of testing among patients [32]. While the acceptance of universal opt-out HIV testing among emergency department patients has varied greatly [33], [34] and was as low as 24% in a recent trial [35], physician recommended HIV testing is more acceptable for most patients [32]. Thus, expanding targeted HIV testing using indicator diseases may be an agreeable approach for patients. In our study, HIV indicator diseases could potentially detect approximately two out of every five persons with HIV at an earlier stage. If the earlier diagnosis leads to earlier ART initiation, indicator disease-based HIV screening has the potential to reduce both HIV-related morbidity and HIV transmission [36], [37], [38]. This screening strategy should of course be added to the usual HIV screening initiatives to ensure un-delayed diagnosis of the remaining 60% of persons with HIV. Almost one-third of cases in our study had no hospital contacts in the 5 years prior to their HIV diagnosis. Therefore, national screening initiatives could aim to expand current non-hospital based strategies such as community outreach programs aimed at high-risk groups (e.g. sex workers and drug users) [39], [40].

In conclusion, knowledge of HIV indicator diseases may optimize national HIV testing programs but the effectiveness of indicator disease-based HIV screening needs confirmation in clinical studies. Although the use of indicator diseases may enhance the identification of undiagnosed HIV-infected individuals, this strategy can only be supplementary to systematic HIV testing of risk groups identified through information about their behavioral risk-taking profiles.

## Supporting Information

Table S1 *International Classification of Diseases*, version 8 and version 10 codes used for the analyses.
(XLS)

Table S2 Section 1 to 22 contains information and risk estimates on subcategorized disease groups for each of the 22 major disease categories. First-time diagnoses within each disease group were recorded if occurring less than 5 years prior to index date. Unadjusted odds ratio [OR] and adjusted odds ratio [aOR] for all other variables within the same disease category are shown with 95% confidence intervals [95% CI]. The number of persons in the analyses were: Cases (n = 2,036) and controls (n = 35,718).
(XLS)
